# Looped Elastic Resistance during Squats: How Do Band Position and Stiffness Affect Hip Myoelectric Activity?

**DOI:** 10.3390/jfmk7030060

**Published:** 2022-08-19

**Authors:** Eduardo C. Martins, Lucas B. Steffen, Diogo Gomes, Walter Herzog, Alessandro Haupenthal, Heiliane de Brito Fontana

**Affiliations:** 1 Biomechanics Laboratory, School of Sports, Federal University of Santa Catarina, Florianópolis 88037-000, SC, Brazil; 2 Human Performance Laboratory, Faculty of Kinesiology, University of Calgary, Calgary, AB T2N 1N4, Canada; 3 Department for Health Sciences, Federal University of Santa Catarina, Araranguá 88900-000, SC, Brazil; 4 Department of Morphological Sciences, School of Biological Sciences, Federal University of Santa Catarina, Florianópolis 88040-900, SC, Brazil

**Keywords:** exercise, functional training, gluteal muscles, rehabilitation, physical therapy, activation, electromiography, gluteus medius, tensor fascia late, exercise prescription

## Abstract

Looped elastic bands around the thigh are commonly used during squats and result in increased hip activation. Due to the closed kinetic chain nature of the squat exercise, one may expect that placing the elastic band on distal segments, close to the floor contact, may not result in the same increase in hip muscle activation as that achieved with a looped band around the thigh. We analyzed the effects of band position (thigh, lower leg, and forefoot) and band stiffness on the myoelectric activity of the tensor fascia latae, gluteus medius, gluteus maximus, biceps femoris, vastus medialis, and vastus lateralis during squats in 35 healthy subjects (18 men and 17 women). The greatest myoelectric activity of hip muscles was observed when the stiffest band was positioned around the forefeet with an increase in 24% for the tensor fascia latae, 83% for the gluteus medius, and 68% for the gluteus maximus compared to free (without resistance band) squatting. Contrary to previous thinking, the use of elastic bands around the forefeet during squats can elicit increased myoelectric activity of hip muscles, with a magnitude often greater than when the band is placed around the thigh segments.

## 1. Introduction

Looped elastic bands around the lower limbs are often used during weight bearing and free weight squat exercises with the aim of enhancing the recruitment of hip muscles and assist in proprioception [1,2,3]. Previous studies testing the effect of adding a looped elastic band around the thigh segments during squats confirmed an increase in hip myoelectric activity. Specifically, increased activation of gluteus maximus (GMax) and gluteus medius (GMed) were observed with the use of elastic bands around the thigh compared to the squat without elastic resistance [1,3,4]. 

Elastic band positioning other than at the thigh for the squat exercise, such as at the ankles or the feet, have not been investigated in the literature and are typically not explored in clinical practice [1,3,4]. A reason for the lack of clinical use of the elastic band at more distal positions during squats has been the assumption that placing the band close to the floor does not increase the demand of the hip muscles to the extent as when placing around the thigh. However, given the double-leg support used during squatting, the dynamics of the exercise cannot be predicted from the kinematics alone. There is an infinite number of possible strategies of muscle activation that can be adopted when adding the elastic band without altering the kinematics of the squat exercise. This flexibility in muscle strategies and ground reaction force production can lead to non-intuitive muscular demands, as has been demonstrated for the footwork exercise in the Pilates reformer [5]. The determination of the effect of different band positions on hip muscles requires direct quantification of muscle activity using electromyography [1,6]. 

In studies investigating changes in myoelectric activity associated with the use of the looped elastic band, the possible effects of band stiffness on muscle activation were ignored [1,2,4], except for a recent study in which an increase in GMax activity during squatting was observed with increased band stiffness (7 N/cm vs. 2 N/cm) [3]. However, a potentially crucial gap for clinical application exists for the combined effects of band stiffness and position. 

The assumption of reduced myoelectric activity of hip muscles for distal (lower leg and forefeet) compared to proximal (thigh) positions for the elastic band may not hold when tested against experimental data. In studies that analyzed the effect of band position on hip muscle activity during the side-stepping exercise—an exercise that involve phases with double leg support and phases with single leg support—it was observed that activation of the gluteal muscles was increased with distal compared to proximal band placement [6,7]. While this finding might seem to be in contrast with the expected reduction in EMG for distal positions, there are substantial differences in movement dynamics between squatting and the side-stepping exercise, with side-stepping characterized by a lateral displacement through steps that involve a single leg support [8], while squatting is performed with double-leg support throughout. No previous studies have been conducted to systematically evaluate the effect of different band positions during the squat exercise. Understanding the effect of elastic band position and stiffness on hip myoelectric activity during the squat exercise may contribute to physiotherapists and athletic trainers’ decision making when prescribing this exercise for therapeutic or training purposes. Therefore, the aim of the present study was to determine the effects of elastic band position and stiffness on the myoelectric activity of GMax, GMed, tensor fascia lata (TFL), biceps femoris (BF), vastus medialis (VM), and vastus lateralis (VL) during the squat exercise. In accordance with the common clinical assumption, we tested the hypotheses that more distal positions of the elastic band (i.e., closer to the floor) result in a reduction in the myoelectric activity of the hip muscles. Furthermore, we also hypothesized that changes in band stiffness have a smaller effect on hip muscle activity when placed more distally (feet, ankle) compared to when placed more proximally (thigh).

## 2. Materials and Methods

### 2.1. Study Design and Participants 

An experimental, laboratory-based study with repeated measures was conducted. The research questions addressed were based on discussions in a weekly community-oriented seminar series where health professionals and students are invited to participate in discussions aimed at translating biomechanical concepts and scientific evidence into clinical/ exercise practice. This seminar series is conjointly organized by academic and non-academic researchers from the Federal University of Santa Catarina (UFSC), the State University of Santa Catarina (UDESC), and the FisioLab Institute in Florianopolis, Brazil. A total of 35 subjects (18 men, age: 25 ± 4 years; height: 1.76 ± 0.10 m; body mass: 82 ± 12 kg and 17 women, age: 26 ± 5 years; height: 1.64 ± 0.07 m; body mass: 59 ± 9 kg) volunteered and gave written informed consent to participate in this study. The inclusion criteria for participation were: (i) age between 18–35 years; (ii) absence of injury of the locomotor system, metabolic diseases and known infectious and/or inflammatory processes; (iii) no history of orthopedic surgery; and (iv) current regular experience in functional resistance exercises with an elastic band (including the squat) for at least 6 months (self-reported). All procedures were approved by the Institution’s Human Research Ethics Committee.

### 2.2. Procedures

Age, sex, dominant lower limb, mass, and height were verified for each subject. Subject’s skin was prepared for surface electromyography, and bipolar electromyography electrodes were positioned on TFL, GMed, GMax, VM, VL, and BF following the Surface Electromyography recommendations for the Non-Invasive Assessment of Muscles (SENIAM). Each subject performed complete squat cycles for 20 s (~5 repetitions) for 10 experimental conditions that were randomly assigned. An interval of at least one minute was enforced between conditions and was extended upon subject request. Conditions consisted of squatting without band (for normalization purposes) and squatting with a combination of band positions and band stiffness. Three band positions were tested: (i) band around the thigh segments (two fingers above the lateral condyles of the femur); (ii) band around the lower leg (two fingers above the lateral malleolus); and (iii) band around the forefoot (at the level of the metatarsophalangeal joints). Each position was tested with three band stiffnesses: low, moderate, and high stiffnesses.

The stiffness of each elastic band was determined experimentally using a load cell mounted in a mechanical testing apparatus with each end of the looped elastic band fixed. Force was measured for steps of 3 cm of elongation from slack length to 60 cm of distance between the fixed ends. Stiffness was calculated as the best fit linear slope of the force-elongation data obtained between 45 cm and 60 cm of length. Before the start of each trial, the length of the elastic band when placed around the subject’s thigh, ankle, and forefeet was measured with a tape and used to estimate the force in the elastic band in the standing position with stance width standardized to the distance between shoulder acromions. Stiffnesses of our elastic bands were approximately 0.6, 1, and 1.7 N/cm. In Table 1, the estimated force of the three elastic bands when placed around the thigh, ankle, and forefeet are shown for the men and women in our sample. 

Squat depth was limited to 90° of knee flexion with a horizontal metal rod being placed behind the subject in such a way that, when the volunteer squatted and touched it, feedback with regards to the depth limit was provided. Stance width was standardized to the distance between shoulder acromions. With this distance marked on the floor, subjects were instructed to place the distal end of their halluxes over each limit of the line. Forefoot alignment was determined based on individual preference. Subjects were instructed to keep the elastic band tensioned throughout the squat cycles, and a familiarization period was allowed. The speed of squatting was controlled using a metronome (50 bpm) and subjects were instructed to match the limit of the movement to the beeps of the metronome. A professional experienced in squat exercises was responsible for evaluating the adequacy of the movement with regards to band tension or squat cadence. A repetition of the attempt was performed if necessary.

### 2.3. Instruments

An 8-channel EMG system (New Miotool, Miotec^®^, Porto Alegre, Brasil), was used at 2000 Hz acquisition frequency. Electromyography signals were corrected by offset and rectified. The linear envelope of the signal was calculated using a 6 Hz 2nd order recursive Butterworth filter. The first and the last repetitions [of the five repeat squats] in each condition were discarded, and the total myoelectric activity, i.e., the integral during the squat for the three central repetitions, was calculated. After calculating the myoelectric activity for each condition, the magnitude was normalized to that obtained during the squat without band. 

### 2.4. Statistical Analysis

Data normality was confirmed using the Shapiro–Wilk test. A mixed-model analysis of variance (α = 0.05) was used, considering the elastic band position and stiffness as repeated measures (three levels for each factor), and sex (men and women) as an independent factor. Bonferroni test with corrections was used for multiple comparisons. The effect sizes were expressed as partial eta-squared (ηp^2^) values within repeated measures (ηp^2^ ≤ 0.01: small effect, ηp^2^ ≤ 0.06: medium effect, ηp^2^ ≤ 0.14: large effect) [9].

## 3. Results

**Figure 1:**
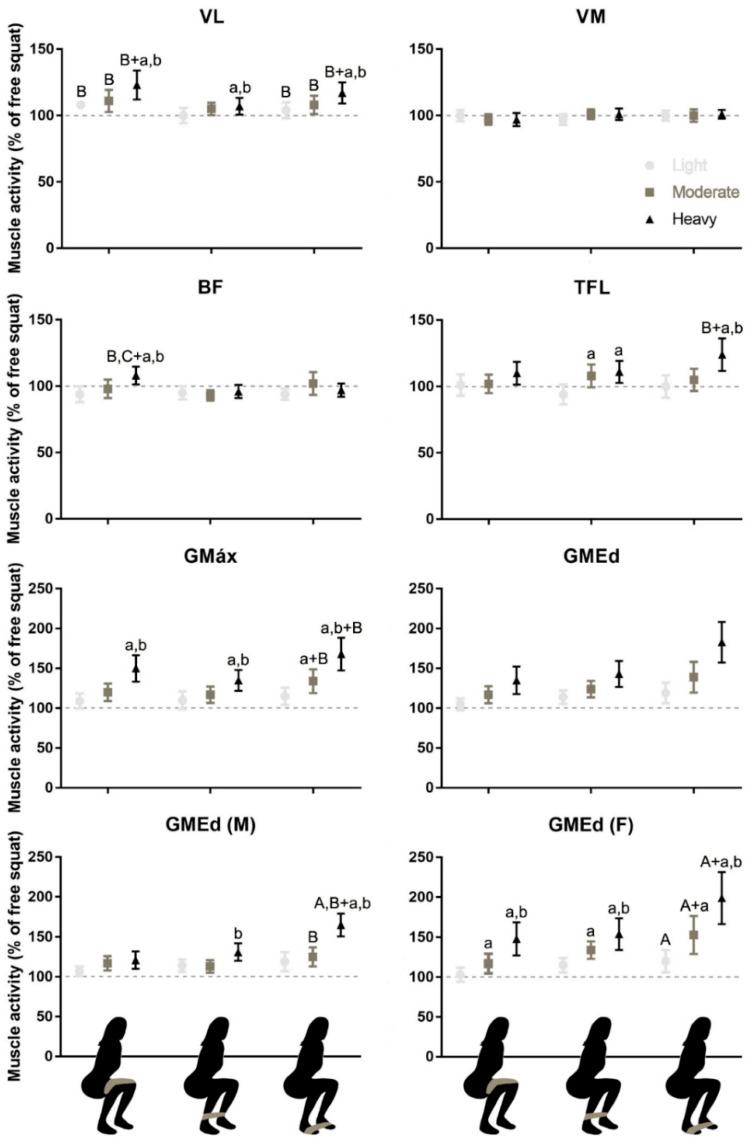
Estimated mean and confidence intervals of the myoelectric activity of vastus medialis (VM), vastus lateralis (VL), biceps femoris (BF), gluteus maximus (GMAX), gluteus medius (GMED), and tensor fascia late (TFL) during squatting for all combinations of band stiffness (light, moderate, and heavy) and band position (illustrated at the bottom) tested in this study. Myoelectric activity measured for the entire squat cycle was normalized to the myoelectric activity obtained in squatting without elastic resistance (100%). Interactions between sex and band stiffness were observed for the GMED, so inferential results are shown for men (M) and women (W) separately. Interactions between the effects of band position and stiffness were observed for the GMED (M), GMAX, TFL and BF muscles. Dashed line indicates the myoelectric activity during the free squat with no band. Small letters (a, b) indicate greater, for a given position, myoelectric activity compared to the light, moderate, and heavy band stiffness, respectively. Capital letters (A, B, C) indicate, for a given stiffness, greater myoelectric activity compared to the band around the thigh, the lower leg, and the forefeet respectively.

The GMed was the only muscle that showed different effects between men and women (double interaction sex*stiffness *p* = 0.008; ηp^2^ = 0.14), with an overall greater effect of stiffness in women compared to men (Table 2). Except for VM, the myoelectric activity during the squat exercise was affected by the elastic band stiffness and/or the position of the band (Figure 1).

For the gluteus medius in men (Figure 1, GMed M), a significant interaction between the effect of band position and the effect of band stiffness was observed (*p* = 0.013; ηp^2^ = 10.56). An increase in myoelectric activity was observed when the band of heavy resistance was moved from the thigh to the ankle (*p* = 0.001) and from the ankle to the forefeet (*p* = 0.001), while the moderate resistance band produced differences only between the ankle and the forefoot position (0.038). No effect of band stiffness was observed for the GMed when the band was placed around the thigh (*p* > 0.05).

For the gluteus medius in women (Figure 1, GMed W), no significant interaction between position and stiffness was observed (*p* = 0.155, ηp^2^ = 4.608). An increase in myoelectric activity was observed when the band (regardless of stiffness) was moved from the thigh to the forefoot (*p* = 0.008, ηp^2^ = 9.807). Additionally, a significant effect of band stiffness was observed at all positions (*p* < 0.001, ηp^2^ = 37.515).

For the gluteus maximus (Figure 1, GMax), a significant interaction between the effect of band position and the effect of band stiffness was observed (*p* = 0.023, ηp^2^ = 11.75). An increase in myoelectric activity was observed when the band was moved from the ankle to the forefoot for the moderate (*p* = 0.002) and heavy resistance (*p* < 0.001) bands. Compared to the bands of light and moderate resistance, the use of a heavy elastic resistance band resulted in a higher myoelectric activity of GMax regardless of band position (*p* < 0.001).

For the tensor fascia latae (Figure 1, TFL), a significant interaction between the effect of band position and the effect of band stiffness was observed (*p* = 0.031, ηp^2^ = 11.01). An increase in myoelectric activity was observed when the band of heavy resistance was moved from the ankle to the forefoot (*p* = 0.045), with no differences in myoelectric activity across positions for the bands of light and moderate resistance (*p* > 0.05). No effect of stiffness was observed for the TFL when the band was placed around the thigh (*p* > 0.05).

For the muscles around the thigh (BF, VL, VM), moving the band distally did not result in increased myoelectric activity or in a greater effect of increasing stiffness of the elastic band (Figure 1). In fact, for the BF, a significant interaction between the effect of band position and the effect of band stiffness was observed (*p* = 0.008, ηp^2^ = 13.02): the myoelectric activity was greater at the thigh position compared to the ankle (*p* = 0.021) and forefoot (*p* = 0.036), and this effect was only present for the band of heavy resistance, with no differences in myoelectric activity across positions for the bands of light and moderate resistance (*p* > 0.05). No effect of stiffness was observed for the BF when the band was placed around the forefoot or ankle (*p* > 0.05).

For the VL, no significant interaction between position and stiffness was observed (*p* = 0.108, ηp^2^ = 6.57). The thigh and forefoot position resulted in greater myoelectric activity than the ankle position (*p* = 0.002; ηp^2^ = 13.81), and an increase in myoelectric activity was observed across bands (*p* < 0.001; ηp^2^ = 19.1). For the VM, no effect of stiffness (*p* = 0.715; ηp^2^ = 0.64) or position (*p* = 0.338; ηp^2^ = 1.86) was observed.

## 4. Discussion

We investigated the effects of band position and stiffness on the myoelectric activity of GMed, GMax, TFL, BF, VM, and VL muscles during the squat exercise. Based on our results, the hypothesis of a reduction in myoelectric activity of the hip muscles during the double leg support squat when the band is moved from the thigh to the feet must be rejected. While there was a considerable variability in the individual responses to the addition of the elastic band during squatting (Table 2), we observed significant effects of band stiffness and position and often interactions. The greatest myoelectric activity for the hip muscles was observed when the stiffest band was positioned around the forefoot with an increase of 24% for the tensor fascia latae, 83% for the gluteus medius, and 68% for the gluteus maximus compared to the free squatting condition. We highlight that none of the hip muscles analyzed showed a reduction in myoelectric activity when the elastic band was moved distally to the feet. This is the first study to show that moving the band distally may be an effective way of increasing the recruitment of hip muscles during squatting.

Previous studies showed that moving the band distally to the feet may be an effective way of increasing the recruitment of hip muscles during the side-stepping exercise [6,7], an exercise in which a subject moves laterally with a looped resistance band around the lower limbs. In contrast to squatting, the side-stepping exercise contains open kinetic chain phases, and therefore, a greater effect of elastic band resistance is expected at the distal (knee/ankle) compared to the proximal (thigh) positions. Interestingly, compared with placing the band around the ankles, placing the band around the feet for resisted side stepping produced greater activity in the gluteal muscles without increasing TFL activity. In our study, for the squat exercise, the response of the TFL depended on the stiffness of the band: an increase in myoelectric activity was observed when the band of heavy resistance was moved from the ankle to the forefoot, but no differences in myoelectric activity across positions were observed for the bands of light and moderate resistance. There seems to be a minimal band stiffness level that is necessary to result in significant changes between positions. It is possible that the dynamic changes that result from moving the light band across positions are too small to require significant changes in myoelectric activity.

While there are no previous studies on the effect of band position during squatting, the observed increase in GMax muscle activation with increasing band stiffness has been previously reported [3] for squatting with a resistance band around the thigh. The reported relative increase in GMax activation (Figure 3 in their paper) was smaller than that observed in the current study, despite the fact that the stiffest band tested was about four times stiffer than the stiffest band in our study. This is likely because subjects were tested during a barbell squat in the previous study, while in our study, subjects performed a free squat. Additionally, Reece et al. (2020) evaluated only the peak (maximum value) of the myoelectric activity, while we quantified changes in myoelectric activity across the entire squat cycle. The increase in TFL activation with increasing stiffness has not been previously described, and we show that it is only significant when the band is positioned around the lower leg or the forefoot, with no effect observed when the band is placed around the thigh.

The changes in hip dynamics for the different band conditions that might explain the observed effects of band positioning and stiffness on hip myoelectric activity in our study are not known. While the activation of gluteal and TFL muscles is required to abduct the hip, the detailed, three-dimensional function of hip muscles is complex and intersegmental torques cannot be predicted during squat without an inverse dynamics approach with independent measures of GRF for each leg. Additionally, important changes in the action of hip muscles occur with the changes in hip flexion/extension, especially with regards to muscle actions in the transverse plane [10,11,12]. Our results describe the changes in myoelectric activity that occur for different band positions and stiffness and show that the assumption of reduced hip myoelectric activity for squatting with the band around a distal body segment (close to the floor), compared to the band around the thigh, is not correct. Future studies evaluating the three-dimensional dynamics of squatting with elastic bands would be instrumental in expanding our understanding regarding the joint dynamics that emerge from the interaction between the elastic band and the floor during squatting [8].

Not all muscles responded to the increase in band stiffness with increased myoelectric activity. The increase in gluteal myoelectric activity observed with the increase in band stiffness contrasts with the small effect observed in our study for the quadriceps muscles (VL and VM). This is likely related to the role of VM and VL as knee extensors—with little function in the frontal or transverse planes, the planes mostly perturbed by the resistance bands [13]. Of all muscles, the BF had the smallest increase in myoelectric activity with the addition of the elastic bands. In fact, values of myoelectric activity smaller than 100% for BF (Table 2) indicate that the addition of an elastic resistance to the squat resulted in a reduction in myoelectric activity compared to the squat without an elastic band. Average values smaller than 100% are observed for the BF in all conditions tested, except for two—which show an average increase of 3 to 8% in BF myoelectric activity. Our results suggest that the addition of elastic bands during the squat exercise are not justified when the aim is to increase BF or quadriceps myoelectric activity.

The reader should be aware of the limitations of EMG when interpreting our results and implications for clinical/ training practice. These have been described in detail elsewhere [14,15,16]. We did not measure squat kinematics in this study. An experienced physiotherapist (L.B.S.) prescribed the exercise, and all subjects were experienced with squat exercises and exercises with elastic bands. In this study, we controlled the distance between the forefeet for all trials collected, but rearfoot alignment was not standardized as we did not want to interfere in the subjects preferred foot alignment. Previous studies have shown that changes in foot alignment result in changes in knee momement [17] and might influence the myoelectric activity of the muscles evaluated in this study. While we did not control for foot alignment, our analysis was based on repetitive measures, which would minimize the implications of a potential confounding effect of foot alignment in the interpretation of our results. Fatigue was not directly evaluated in this study, and the resistance of the band was tested prior to but not after data collection. However, testing was performed using a randomized order, thereby guarding against a systematic effect of fatigue confounding the results. Furthermore, repeat testing of the band stiffness resulted in a virtually perfectly elastic behavior with no measurable difference between trials, suggesting that band stiffness was likely not changed during the test protocol. Nevertheless, not evaluating fatigue explicitly and not testing the stiffness of the elastic bands before and after each use must be considered a limitation of the experimental approach.

## 5. Conclusions

We conclude, based on our findings, that more distal positions of elastic resistance bands along the lower limb during squat result in an increase, and not a decrease, in the myoelectric activity of hip muscles. We further conclude that changes in band stiffness have a greater effect on hip muscle recruitment when positioned at distal compared to proximal sites of the leg. Squatting with an elastic band around the forefoot elicited the greatest myoelectric activity of the gluteal and TFL muscles. Finally, while TFL, gluteus medius, and gluteus maximus myoelectric activity was sensitive to changing the elastic band stiffness and position, VL, VM, and BF were not.

## Figures and Tables

**Table 1:** Mean (and the standard deviation) for the elastic band force (in N) when stretched around each position tested subjects’ segment.

Position	Sex	Light	Moderate	Heavy
**Thigh**	M	53.5 (5)	90 (8.5)	141.5 (13.5)
	F	48 (2)	80.5 (3.5)	126.5 (5.5)
	M + F	51 (4.5)	85.5 (8)	134 (13)
**Lower leg**	M	50 (5.5)	83.5 (9.5)	131 (14.5)
	F	44.5 (3.5)	74 (6)	116 (9.5)
	M + F	47.5 (5.5)	80 (9)	124 (14.5)
**Forefeet**	M	60.5 (6.5)	101.5 (11)	159.5 (18)
	F	50.5 (3)	85 (5)	133 (8)
	M + F	55.5 (7)	93.5 (12)	147 (19.5)

**Table 2:** Sample mean (standard deviation) of vastus medialis (VM), vastus lateralis (VL), biceps femoris (BF), gluteus maximus (GMAX), gluteus medius (GMED) for men (M) and women (W), and tensor fascia lata (TFL) during squatting for all combinations of band stiffness (light, moderate, and heavy) and band position (thigh, lower leg, and forefeet) tested in this study. Myoelectric activity measured for the entire squat cycle was normalized to the myoelectric activity obtained for squatting without elastic resistance and is shown in %. Results are shown for male (M) and female (F) subjects as well as for the total sample pooled across sexes (M + F).

Muscle	Sex	Thigh			Lower Leg			Forefeet		
		Light	Moderate	Heavy	Light	Moderate	Heavy	Light	Moderate	Heavy
TFL	M	99 (22)	101 (23)	106 (29)	89 (25)	104 (29)	106 (27)	99 (25)	99 (22)	116 (39)
	F	102 (26)	102 (23)	113 (24)	99 (19)	112 (23)	116 (23)	101 (27)	111 (28)	131 (34)
	M + F †	101 (24)	102 (21)	110 (26)	94 (23)	**108 ^a^ (26)**	**111 ^a^ (25)**	100 (26)	105 (25)	**124 ^B+a,b^ (37)**
GMed *	M †	107 (18)	117 (27)	121 (33)	114 (23)	113 (24)	**131 ^b^ (33)**	**119 (36)**	**125 ^B^ (36)**	**165 ^A,B+a,b^ (43)**
	F	103 (27)	**117 ^a^ (37)**	**148 ^a,b^ (63)**	115 (28)	**134 ^a^ (33)**	**154 ^a,b^ (60)**	**120 ^A^ (43)**	**153 ^A+a^ (72)**	**199 ^A+a,b^ (98)**
	M + F	105 (23)	117 (32)	135 (52)	114 (26)	124 (31)	143 (49)	119 (39)	139 (58)	183 (77)
GMax	M	104 (31)	123 (35)	142 (59)	102 (21)	111 (22)	130 (20)	108 (18)	123 (25)	161 (46)
	F	113 (27)	117 (32)	158 (41)	118 (43)	122 (38)	140 (52)	122 (42)	144 (57)	175 (75)
	M + F †	109 (29)	120 (33)	**150 ^a,b^ (50)**	110 (34)	117 (31)	**135 ^a,b^ (39)**	115 (33)	**134 ^a,b^ (45)**	**168 ^B+a,b^ (62)**
VM	M	97 (13)	95 (14)	97 (12)	97 (11)	101 (9)	101 (9)	102 (10)	99 (11)	102 (9)
	F	103 (13)	98 (17)	98 (17)	97 (14)	100 (13)	101 (16)	98 (12)	100 (16)	100 (11)
	M + F	100 (13)	97 (12)	97 (15)	97 (12)	101 (11)	101 (13)	100 (11)	100 (14)	101 (10)
VL	M	110 (31)	112 (30)	116 (36)	104 (20)	110 (16)	105 (18)	106 (21)	106 (23)	116 (26)
	F	107 (14)	111 (20)	129 (29)	97 (14)	100 (11)	108 (19)	102 (16)	110 (19)	117 (23)
	M + F	**108 ^B^ (2)**	**111 ^B^ (25)**	**123 ^B+a,b^ (33)**	100 (17)	105 (14)	**107 ^a,b^ (19)**	**104 ^B^ (18)**	**108 ^B^ (21)**	**117 ^B+a,b^ (24)**
BF	M	94 (22)	100 (25)	111 (25)	93 (13)	92 (11)	95 (16)	98 (14)	98 (23)	95 (11)
	F	94 (13)	97 (16)	105 (14)	97 (17)	94 (12)	96 (15)	91 (12)	106 (29)	99 (17)
	M + F †	94 (18)	98 (21)	**108 ^B,C+a,b^ (20)**	95 (15)	93 (12)	96 (15)	94 (13)	102 (26)	97 (15)

* Interaction between sex and stiffness; † interaction between band stiffness and position; superscript small letters (a, b) indicate greater myoelectric activity compared to the light, moderate, and heavy band stiffness, respectively; superscript capital letters (A, B, C) indicate greater myoelectric activity compared to the band around the thigh, the lower leg, and the forefeet, respectively.

## Data Availability

Data can be made available upon request.
